# Quantitative Approach Based on Wearable Inertial Sensors to Assess and Identify Motion and Errors in Techniques Used during Training of Transfers of Simulated c-Spine-Injured Patients

**DOI:** 10.1155/2018/5190693

**Published:** 2018-03-05

**Authors:** Karina Lebel, Vanessa Chenel, John Boulay, Patrick Boissy

**Affiliations:** ^1^Faculty of Medicine and Health Sciences, Orthopedic Service, Department of Surgery, Université de Sherbrooke, 3001 12e Avenue Nord, Sherbrooke, QC, Canada J1H 5N4; ^2^Research Center on Aging, 1036 Belvédère Sud, Sherbrooke, QC, Canada J1H 4C4; ^3^Interdisciplinary Institute for Technological Innovation (3IT), Université de Sherbrooke, 3000 Université Blvd, Sherbrooke, QC, Canada J1K 0A5; ^4^DESS-Thérapie du Sport, Université du Québec à Trois-Rivières, 3351 Boul. des Forges, C.P. 500, Trois-Rivières, QC, Canada G9A 5H7; ^5^Exercise Science/Athletic Therapy, Concordia University, 7141 Sherbrooke St. W., Montreal, QC, Canada H4B 1R6

## Abstract

Patients with suspected spinal cord injuries undergo numerous transfers throughout treatment and care. Effective c-spine stabilization is crucial to minimize the impacts of the suspected injury. Healthcare professionals are trained to perform those transfers using simulation; however, the feedback on the manoeuvre is subjective. This paper proposes a quantitative approach to measure the efficacy of the c-spine stabilization and provide objective feedback during training. *Methods*. 3D wearable motion sensors are positioned on a simulated patient to capture the motion of the head and trunk during a training scenario. Spatial and temporal indicators associated with the motion can then be derived from the signals. The approach was developed and tested on data obtained from 21 paramedics performing the log-roll, a transfer technique commonly performed during prehospital and hospital care. *Results*. In this scenario, 55% of the c-spine motion could be explained by the difficulty of rescuers to maintain head and trunk alignment during the rotation part of the log-roll and their difficulty to initiate specific phases of the motion synchronously. *Conclusion*. The proposed quantitative approach has the potential to be used for personalized feedback during training sessions and could even be embedded into simulation mannequins to provide an innovative training solution.

## 1. Introduction

The majority of spinal cord injuries (SCI) occur at the cervical level (c-spine) and are caused by traumatic events (traffic accident, sports, and falls) [1]. When a person is suspected of having a SCI, the current guidelines recommend spinal motion restriction of the head and spine when transferring and transporting the individual [2–4]. Nevertheless, it is estimated that 3%–25% of neck-injured patients experience neurological deterioration during patient management [5, 6]. Throughout that continuum of care for suspected SCI, prehospital and hospital allied health professionals have to perform multiple transfers of the SCI patient during which the c-spine must be stabilized adequately to limit motion and minimize the consequences of the suspected injury. In research context, efficacy of different transfer techniques has been studied [5, 7, 8]. Regardless of the results, the log-roll remains the current standard of care in a prehospital management context, mainly because of its applicability to a wide variety of contexts (prone and supine patients) and the limited minimum number of paramedics required to perform the technique [4]. Briefly, the log-roll requires a rescuer positioned at the patient's head to manually maintain inline stabilization of the head to the trunk while the patient is rolled on his or her side. Health professionals are trained by means of simulation to stabilize the c-spine and perform these transfers using different c-spine stabilization techniques and equipment. But as described by Del Rossi et al. [9], the log-roll is a complex manoeuvre requiring to move in an appropriate arc of circle, while at the same time considering the possible dynamic change in spine slope. This change in spine slope can occur during the manoeuvre due to the patient's body proportions (i.e., when rolling the patient on his/her side, the width of the patient's shoulders and hips may cause a change in spine angle, relative to the floor). Hence, training is critical to ensure the best possible c-spine stabilization. A typical training session uses a simulated patient on which the manoeuvre is performed under the supervision of a qualified trainer. Feedback on the quality of the stabilization manoeuvre is subjectively given by the simulated patient and/or the expert. Considering the proven limited ability of rescuers and simulated patients to adequately judge the performance of stabilization manoeuvres [10], the demonstrated variability in stabilization performance between rescuers [9, 11–13], and the described complexity of the manoeuvre [9], there is a critical need to develop objective and meaningful feedback measures for c-spine stabilization techniques during training scenarios.

Lately, wearable inertial systems such as attitude and heading reference systems (AHRS) have emerged as an alternative for motion capture systems. Inertial systems are a portable, flexible, and relatively low-cost alternative that is not affected by visual occlusions, offering new possibilities for in-context measurement of kinematic features [14]. AHRS have already been used successfully to assess the performance of different c-spine stabilization techniques [10–12]. Furthermore, the interest of trainers and trainees to use such technology to provide visual objective feedback was also explored by our team in a nursing context and shown to be very promising [15]. Building on these results, the goal of the current research program is therefore to develop a measurement approach to objectively assess c-spine motion in diverse situations during c-spine stabilization training and to allow for constructive feedback to be given to the trainee. The current paper first describes a proposed methodology which allows for visual and numerical feedback during c-spine transfers and then proposes a set of explicit, meaningful, and user-friendly feedback indicators of performance with regard to a simulated c-spine transfer. The use of the method and its discriminative power to assess the performance of the transfer and to understand the possible cause for the undesirable motion are also investigated using a transfer simulation scenario performed by paramedics.

## 2. Material and Methods

### 2.1. Measurement System

**Figure 1:**
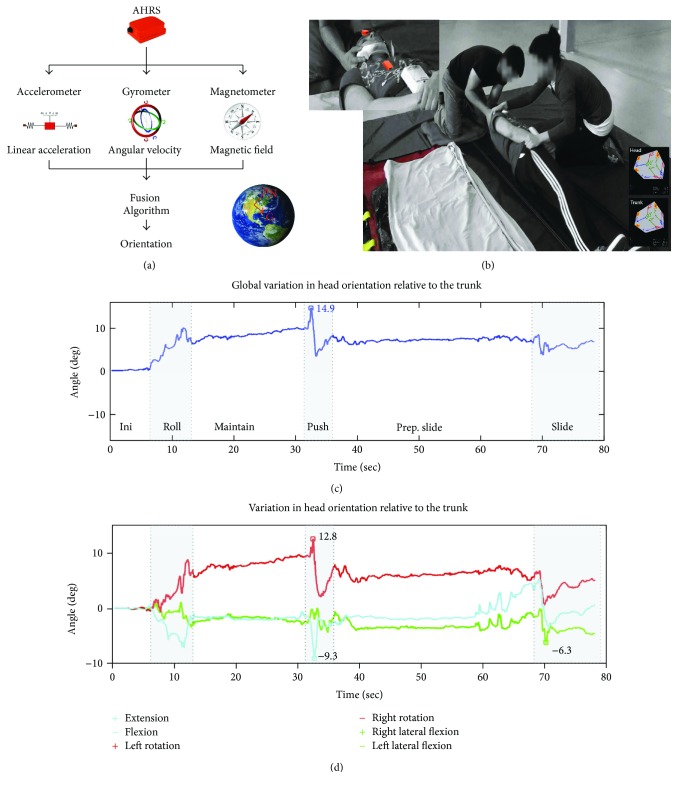
Methods of measurement. (a) Attitude and heading reference systems (AHRS) include a 3-axis accelerometer, gyroscope, and magnetometer to measure, respectively, linear acceleration, angular velocity, and magnetic field. Data are passed on to a proprietary fusion algorithm (included in the measurement system) to estimate the orientation of the platform in a fixed and global reference frame-based gravity and magnetic north. (b) Attached on the forehead and the trunk of a simulated patient, AHRS estimates the orientation of both segments in the same global reference frame. (c) Relative orientation of the head to the trunk can therefore be computed directly from the measurement system. (d) Relative orientation is also decomposed into anatomic motion using a dynamic anatomical alignment process.

The proposed method of measurement is based on wearable inertial sensors, also called attitude and heading reference systems (AHRS), placed on the forehead and the trunk of a simulated patient (SP). SP here refers to an uninjured individual playing the role of a patient with suspected c-spine injury. AHRS are a specific type of inertial measurement unit comprised of 3-axis accelerometers (measuring linear acceleration), gyroscopes (measuring angular velocity), magnetometers (measuring magnetic field), and a fusion algorithm (estimating the orientation of the platform in a fixed and global reference frame) (Figure 1(a)).

AHRS modules on the forehead and the trunk of a simulated patient therefore enable orientation tracking of both the head and the trunk in the same global reference frame (Figure 1(b)). Relative orientation of the head to the trunk can then be mathematically derived (Figure 1(c)). The total movement can also be decomposed into three planes of anatomic motion (sagittal: flexion-extension, coronal: right and left lateral flexion, and transverse: right and left lateral rotation) to enhance comprehension of the measured motion (Figure 1(d)). Decomposition is based on a dynamic anatomical alignment process performed at the beginning of the session. Briefly, a series of unidirectional passive head motions guided by a qualified clinician (flexion-extension, right-left rotations, and right-left lateral flexion) are performed while the data from the two AHRS modules are recorded. Analysis of the change in relative orientation of the head to the trunk during each of these trials allows one to estimate the rotation axis corresponding to each plane of motion. A normalized anatomical alignment matrix is then estimated based on these results. Validation of the anatomical alignment is performed by analysis of the residual motion on each of the calibration trials. Such process allows one to represent the globally defined orientation measured during each test trial in an easier format for clinical interpretation.

The selected AHRS modules for this specific study are the MTx from Xsens Technologies (Netherlands) [16]. Under controlled conditions of motion, MTx modules were shown to have a mean root-mean-squared dynamic accuracy varying between 0.3° and 1.0° for absolute and relative orientation measures under varying conditions of motion when assessed in comparison to an optical motion capture system [17].

### 2.2. Simulation Scenario

**Figure 2:**
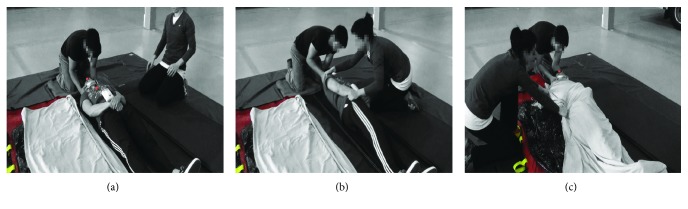
Transfer scenario. (a) The lead rescuer immobilizes the head (initial phase). (b) On signal, rescuers roll the patient on his side (roll phase) and maintain the patient in this position while the assistant pulls the vacuum mattress close to the patient (maintain phase) and slowly rolls the patient back onto the mattress (push phase). (c) The final positioning of the patient into the middle of the mattress is performed by pulling gently on the sheet placed onto the mattress.

The chosen simulation scenario corresponds to the recommended transfer technique performed in prehospital settings in the province of Québec, Canada, and involves transferring the patient onto a vacuum mattress using a log-roll [18]. Each trial was initiated with a fully cooperative simulated patient (SP) lying supine on the floor with a cervical collar and vacuum mattress positioned to the right of the SP. The transfer motion occurs in five phases (Figure 2). In phase 1 (initial phase), the lead rescuer immobilizes the head of the patient using either the head squeeze (HS) or the trap squeeze (TS) method. In brief, HS involves holding of both sides of the patient's head and trying to synchronize the motion of the head to the motion of the trunk during the transfer. With TS, the lead paramedic holds on to the patient's upper shoulders and squeezes the head with his forearms during the transfer. In phase 2 (roll phase), the lead rescuer gives a signal and the patient is rolled on his side with the help of one or more assistant. In phase 3 (maintain phase), the lead rescuer maintains the relative orientation of the head to the trunk as constant as possible while the assistant pulls the vacuum mattress closer to the patient. In phase 4 (push phase), the lead rescuer gives the signal to initiate the slow push of the patient towards the floor and onto the mattress. In the last phase (phase 5: slide phase), the patient is repositioned into the middle of the mattress by pulling carefully onto the sheet placed onto the mattress while the lead rescuer maintains stabilization. The simulation scenario was considered complete when the paramedics confirmed accurate positioning of the SP.

### 2.3. Performance Outcome and Quality Indicators

Throughout the continuum of care, the current clinical guidelines recommend to stabilize the head-to-trunk alignment in order to minimize the possible consequences of a c-spine injury. As such, the main efficacy outcome proposed for performance assessment of c-spine transfer is the peak relative motion, derived from the global change in orientation of the head relative to the trunk (see Figure 1(c)), assessed during each trial. This outcome corresponds to the maximum head/trunk misalignment reached during the entire transfer, regardless of the anatomical direction of that misalignment. Initial interpretation of the relative motion produced can then be done using the anatomically referenced motion (see Figure 1(d)). Although it is useful to get a better image of the direction in which the undesired motion was performed, such representation does not give any insights into the causes for that undesired motion. This level of analysis of the motion can be accomplished in a user-friendly approach with the help of a graphical 2D approach for motion representation and a set of the so-called quality indicators. These quality indicators aim at identifying specific areas for improvement. It should be noted that the proposed indicators are specific to the execution of the log-roll.

**Figure 3:**
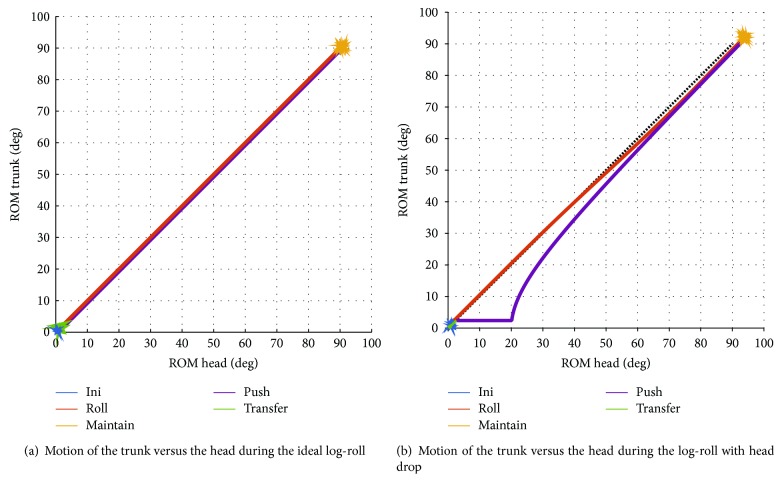
2D graphical representation of a log-roll. Motion of the trunk compared to that of the head during a simulated log-roll for (a) close-to-perfect conditions and (b) head drop during the roll and readjustment at the end of the push.

Characterization of the quality of the manoeuvre is based on the analysis of the temporal and spatial gap between the actual manoeuvre and the ideal representation of the motion during a log-roll. In a perfect log-roll, both head and trunk segments move at the same time (i.e., temporal synchronicity), following concordant paths or “en-bloc” (i.e., spatial synchronicity). The global change in orientation measured for each segment should therefore be the same throughout the manoeuvre. This concept of an ideal log-roll can be modelled using a straight line along the line of identity (slope = 1) on a 2D graph illustrating the change in global orientation measured at the trunk versus the one measured at the head (Figure 3(a)). We hypothesized and verified through simulation that unwanted motion (e.g., head drop in extension during the roll) will be captured in the global change in orientation experienced by the head and would therefore affect the appearance of the graph, as shown in Figure 3(b). Temporal and spatial quality indicators were therefore derived from this 2D graphical representation. The selected indicators are described below, along with their rational.

#### 2.3.1. Temporal Indicators

Temporal synchronization refers to the ability of the rescuers to move the trunk and the head at the same time. Poor communication between the rescuers or difficulty initiating and maintaining smooth motion of the trunk during the roll and push phases (e.g., due to a lack of strength with a large SP) may cause a delay between the motion of the head and the motion of the trunk. Such delay will therefore be investigated. Technically, delays are calculated using both orientation signals (head and trunk) and filtered out using a low-pass 3rd-order Butterworth filter set at a cut-off frequency of 2 Hz. The filter's cut-off frequency was determined through a residual analysis process, using an acceptable residual accuracy threshold. The residual accuracy threshold refers to the ability of the system to detect movement initiation. It is therefore set according to both the measurement system orientation stability and the initial movement stability (i.e., the stability of the head during initial stabilization). In this specific case, a threshold of 0.1° was determined to be adequate from visual observation of a subset of the available trials. Segments are then considered to be in motion as long as their orientation remains above the predefined threshold. Time points at which the roll and push motions are initiated and terminated are then identified for each segment, and the delay values are derived from it.

#### 2.3.2. Spatial Indicators

Spatial synchronicity refers to the idea that both segments move along a proportional arc of circle during both the roll and the push phases, represented by the desired line of identity on the 2D motion graph. Potential spatial quality indicators were therefore developed based on the assumptions regarding ideal log-roll and its representation using a 2D graph of the motion (Figure 3). As such, the deviation from the desired line of identity during the roll and the push phases is investigated as a possible indicator, using the roll and the push best-fit line determined by a least-square approach. An efficient log-roll also assumes that the lead rescuer and his assistant have sufficient control over the motion. Perfect control will allow the rescuers to follow the same path throughout the roll and the push phases. Thus, it is hypothesized that the larger the spread between the two curves on the motion phase plane, the worse the control and result. One way of capturing that “spread” is by calculating the area between the curves. To do so, each trial is divided into two parts: part 1 covering from the initiation of the trial to half-way in the maintain phase and part 2 covering from that point on to the end of the trial. For each part of the signal, the best-fit 4th-order polynomial is identified and the area under the curve produced by that polynomial during the relevant timeframe is evaluated. The area between the curves (ABC) then corresponds to the difference between those areas.

Overall, a performance measure and seven quality indicators of the log-roll transfer are proposed and summarized in Table 1.

### 2.4. Validation of the Proposed Indicators

#### 2.4.1. Participants

In a recent study [11], we compared the efficiency of two stabilization techniques, the head squeeze and the trap squeeze (HS versus TS) to perform a log-roll with two or four paramedics (LR_2_ versus LR_4_) in order to transfer a supine patient onto a vacuum mattress. Each condition was repeated five times. Further details on the protocol are available in [11]. The proposed objective feedback method is based on a secondary analysis of this set of data. Twenty-one paramedics (mean age = 37, range: 22–62) from the Montreal region, Canada, participated in the study after giving their written informed consent following the procedure approved by the Research Ethics Committee of the Jewish General Hospital (Montreal, Canada). Slightly more than half (52%) of the participants were female, and all had the experience in cervical spine management (median = 8 years, range: 1–36 years).

#### 2.4.2. Statistical Analysis

Assessment of the usefulness of the proposed performance measures and quality indicators was performed using all recorded trials from this dataset, regardless of the transfer or immobilization technique used (i.e., LR2_2_ and LR_4_ and TS and HS). For each trial, the performance measure and quality indicators listed in Table 1 were computed using MATLAB version 8.5 (Mathworks) and the values over the five similar trials (i.e., the same technique and number of assistants) were averaged for each participant. Potential explanatory metrics were first identified through univariate regressions, using a baseline threshold of 0.2 for Pearson's correlation coefficient (i.e., |*r*| ≥ 0.2). Assumptions for linearity, independence of residuals, homoscedasticity, multicollinearity, outliers, and normality were all verified. A hierarchical multiple regression approach was then used to determine a model allowing one to predict the main performance measure (relative motion), in which the model was then adjusted for potential confounding factors, namely, the immobilization technique and transfer method [19]. All statistical analyses were performed using SPSS [20].

## 3. Results

### 3.1. Performance Assessment

**Figure 4:**
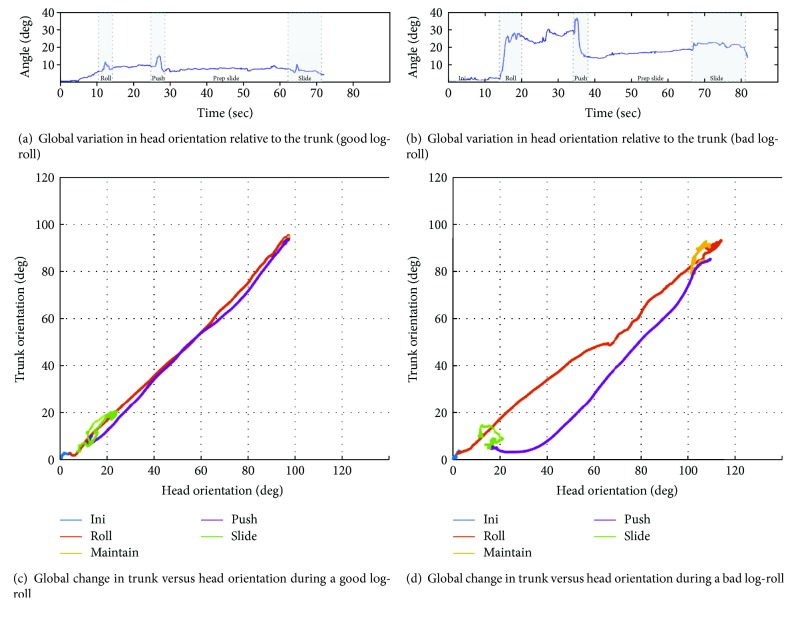
Graphical representation of a good and a bad log-roll. (a, b) Variation in angular motion of the head relative to the trunk during a good (a) and a bad (b) log-roll. (c, d) 2D motion representation of the same good (c) and bad (d) trial.

A global analysis of all trials performed by the 21 paramedics revealed a mean relative peak motion of 22.0° ± 6.5°. Figures 4(a) and 4(b) show the examples of the recorded relative orientation for both a well-conducted log-roll and a poorly conducted log-roll. Overall, the performance of the paramedics varied between 9.5° and 40.8°.

### 3.2. Visual Log-Roll Quality Assessment

2D graphical representation of the log-roll motion provides an in-depth visual representation of each segment during the transfer. Figures 4(c) and 4(d) show the graphical representation of a well-conducted and a poorly conducted trial (as assessed with the global peak motion illustrated in (a) and (b)). As expected, the well-conducted trial representation is close to that of the desired line of identity introduced in Figure 3, while the poorly conducted trial shows diverging curves. In the poorly conducted trial, one can immediately see that the initiation, the roll, and the maintain phases (i.e., the first part of the trial) were close to the desired line of identity and that the problem occurred during the push phase. Indeed, for this specific trial, the 2D graph reveals that the trunk was released faster than the head. The “S”-shaped curve illustrates that once the deviation occurred, the lead rescuer did not make the correction until the entire push phase was almost completed.

### 3.3. Indicators of the Quality of the Manoeuvre

Table 2 presents the mean values as well as the observed range of values obtained for each of the indicators over the 84 cases obtained (i.e., 21 participants, 4 cases/participant including HS and TS techniques, performed with 2 or 4 rescuers). Univariate regressions identified Delay_push_ini_, Slope_Roll_, Slope_Push_ and ABC_Roll-Push_ as potential explanatory variables with Pearson's correlation coefficient *r* ≥ 0.2 (Delay_push_ini_: *r* = 0.442, Slope_Roll_: *r* = 0.462, Slope_Push_: *r* = 0.500, and ABC_Roll-Push_: 0.435). However, an observed significant correlation between Slope_Roll_ and Slope_Push_ (*r* = 0.884) motivated the removal of Slope_Push_ from the list of potential explanatory variables based on phase analysis results which revealed that most of the motion occurs during the roll phase. Next, a hierarchical multiple regression was conducted to evaluate whether Delay_push_ini_, Slope_Roll_, and ABC_Roll-Push_ were all necessary to predict peak motion. Results showed that all indicators were significant. Furthermore, multiple regression assumptions were all met. Linearity was assessed by partial regression plots and a plot of studentized residuals against the predicted values. Independence of residuals was confirmed by a Durbin-Watson statistic of 2.07. Homoscedasticity was established by visual inspection of a plot of studentized residuals versus unstandardized predicted values. There was no evidence of multicollinearity, and a single outlier was removed as it showed a studentized residual of 3.25. Finally, normality was met, as assessed by the Q-Q plot.

Details of the incremental model are shown in Table 3. The model to predict peak motion with Delay_push_ini_, Slope_Roll_, and ABC_Roll-Push_ was statistically significant (*F*(3,79) = 27.983, *p* < 0.001) with 51.5% of the variance in peak motion explained by the model. Once adjusted for the technique and number of assistants, the model remained statistically significant (*F*(5,77) = 18.913, *p* < 0.001) and the variance explained increased to 55.1%.

## 4. Discussion

This study describes a method based on objective measurement of head motion relative to the trunk using AHRS to provide visual and numerical feedback on the performance and quality of c-spine immobilization during log-roll simulation training. Using data from a sample of paramedics performing this specific technique, we used peak motion to assess the variation in the performance of the manoeuvre. In this specific analysis, the identified averaged peak motion of 22.0° was computed over all trials, regardless of the immobilization technique used or the number of assistants. Furthermore, the performance varied between 9.5° and 40.8° among the trials, demonstrating a clear need for objective training. The choice of pooling all trials together for this analysis was based on the fact that regardless of the technique or the number of assistants used, the desired optimal goal of the manoeuvre remains the same: to limit the motion of the head relative to the trunk during the transfer. Details regarding the performance of each technique and number of assistants may be found in [11].

The method introduced herein proposes the use of a 2D graphical approach to assess, at a glance, the quality of the manoeuvre performed at each segment level. As such, simulated 2D graphical representation of well-conducted and poorly conducted trials was confronted to actual good and bad trials and was shown to be concordant. Although the usefulness of such graphic was not directly tested with the paramedics, it has face validity in the fact that it directly illustrates where and when excessive motion occurred, with minimal explanation of how an ideal trial would appear.

The 2D graphical representation of motion was also used as the baseline for the definition of the potential quality indicators, in both the temporal and the spatial domains. For the specific context tested, a univariate regression model revealed that Delay_push_ini_ has a significant relationship to peak motion. In all cases, the greater the delay, the greater the peak motion (i.e., the worse is the performance). Delay at initiation of the motion for the roll phase was not shown to be significant, and therefore the relationship between the communication and timing does not seem to be the major issue. Univariate regression also showed that all the three spatial indicators proposed (Slope_Roll_, Slope_Push_, and ABC_Roll-Push_) have a moderate linear relationship with the recorded peak motion. The larger the deviation of the estimated slope from the ideal, the worse the performance. Similarly, the smaller the area between the roll and the push curves, the better the performance. This is consistent with the hypothesis that paramedics with sufficient control over the motion will more likely execute an efficient log-roll. Control may be influenced by different factors, including training and force. The limit to this study is that the participants' force was not measured. It is therefore impossible, at this stage, to evaluate the impact of strength on the results. The multiple regression model identified the roll slope, the spread between the roll and the push curves (ABC_Roll-Push_), and the delay at push initiation as the main explanatory variables associated with the log-roll performance which explained 52% of the variance. Adding the technique and the number of assistants in the model increased that rate to 55% of the variance explained with the model.

The chosen statistical analysis considered the different parameters as absolute values as the purpose was to look at the influence of synchronicity (temporal and spatial) on the quality of the manoeuvre performed. However, with regard to providing constructive feedback to healthcare professionals, the sign of the delay and its slope difference are also interesting as they reveal which segment moved before or faster than the other. For example, a negative delay at push initiation reveals that the trunk started earlier than the head for that specific phase.

The proposed set of quality indicators is a first attempt at characterizing the quality of a log-roll manoeuvre from a motion coordination-based point of view. The authors feel that this level of feedback can be sufficient in improving the technique during training sessions. However, we recognize that not all specific cases can be assessed using those indicators and that a more in-depth analysis would be required to do so. For example, oscillations during the motion will not be captured directly with the current set of indicators but will appear in the 2D motion graphical representation and could be addressed as such during debriefing. Thus, this study is considered the first step towards the general objective to develop a simulation-based tool to objectively assess c-spine motion.

Furthermore, the techniques used to derive the indicators are straightforward. Although advanced techniques could be investigated to improve, for example, the accuracy of the delay at initiation, it shall be kept in mind that the goal of the present project is to provide a quick and user-friendly approach to assess c-spine motion in training scenarios and provide efficient real-time feedback on the manoeuvre performed in order to improve the technique. As such, the proposed indicators with their current accuracy were able to explain 55% of the variation in performance observed in the study results and are therefore believed to be sufficient to provide an efficient feedback to the trainee.

Indicators were developed using the data from wearable AHRS. Wearable AHRS offer accurate orientation measurements that capture motion of the head relative to the trunk in relatively constraint-free conditions at an affordable price compared to more traditional motion capture technology. Their form factor allows researchers to collect data in varying clinical context scenarios, avoiding problems such as positioning limitations that might occur when an optical marker is blocked from the camera lens view. It shall, however, be noted that the developed indicators appear to be independent of the motion capture measurement system and could therefore be used with other systems such as magnetic motion capture systems [21]. The measurement approach proposed could also even be embedded into simulation mannequins to provide an innovative and complete training solution. A simpler version of this objective feedback training concept using a mannequin and AHRS was already tested in a training scenario with nursing students and perceived as useful and stimulating [15]. The next logical step of this study is therefore to address the usefulness of the proposed complete approach (graphical representation and quality indicators) in a specific training context to evaluate the capability of this methodology to provide meaningful feedback to improve technique performance.

## 5. Conclusions

This study proposes a methodology which provides objective feedback to participants during c-spine transfer simulated scenarios using AHRS. The concept was applied to the specific case of prehospital transfers for which three specific metrics (slope of the motion performed by the trunk compared to the motion performed by the head during roll, delay at push initiation, and area between the roll and the push phases) have been identified as explaining 55% of the performance variation when combined with the technique and number of assistants used. The proposed approach has a potential to be used for personalized feedback during training and could even be directly embedded into simulation mannequins in order to provide a complete training solution.

## Figures and Tables

**Table 1:** Performance and quality indicators for the log-roll.

Category	Indicator	Equation	Description
Performance measure	ROMrel_peak_	Max(ROMrel)	Peak change in global orientation of the head relative to the trunk

Temporal quality indicators	Delay_roll_ini_	|t_Head_Roll_ini__ − *t*_Trunk_Roll_ini__|	Delay at roll initiation
	Delay_roll_end_	|*t*_Head_Roll_end__ − *t*_Trunk_Roll_end__|	Delay at roll termination
	Delay_push_ini_	|*t*_Head_Push_ini__ − *t*_Trunk_Push_ini__|	Delay at push initiation
	Delay_push_end_	|*t*_Head_Push_end__ − *t*_Trunk_Push_end__|	Delay at push termination

Spatial quality indicators	Slope_Roll_	|*m*_roll_ − 1|	Difference between the slope of the best-fit line of the roll curve and the ideal line of identify
	Slope_Push_	|*m*_push_ − 1|	Difference between the slope of the best-fit line of the push curve and the ideal line of identity
	ABC_Roll-Push_	|AUC_roll_ − AUC_push_|	Area contained between the curves from the roll and the push phases

**Table 2:** Recorded values for all potential quality indicators for the log-roll.

Category	Indicator	Mean (Std Dev)	Range (min, max)
Performance measure	ROMrel_peak_	22.0° (6.5°)	(9.5°, 40.8°)

Temporal quality indicators	Delay_roll_ini_	0.20 s (0.14 s)	(0.02 s, 0.76 s)
	Delay_roll_end_	0.29 s (0.24 s)	(0.00 s, 1.12 s)
	Delay_push_ini_	0.10 s (0.15 s)	(0.00 s, 0.86 s)
	Delay_push_end_	0.18 s (0.17 s)	(0.00 s, 0.94 s)

Spatial quality indicators	Slope_Roll_	0.12 (0.08)	(0.03, 0.34)
	Slope_Push_	0.11 (0.08)	(0.02, 0.36)
	ABC_Roll-Push_	396.6 (219.8)	(47.8, 1104.1)

**Table 3:** Hierarchical multiple regression predicting peak relative motion from Slope_Push_, ABC_Roll-Push_, and Delay_push_ini_.

Variable	Peak motion			
	Model 1		Model 2	
	*B* ^1^	*β*	*B* ^1^	*β*
Constant	12.053		17.63	
Slope_Roll_	35.797	0.426	31.77	0.378
ABC_Roll-Push_	0.011	0.383	0.01	0.400
Delay_Push_ini_	12.388	0.286	10.18	0.235
Technique	—	—	−0.96	−0.076
Number of assistants	—	—	−2.43	−0.191
*R* ^2^	0.515		0.551	
*F*	27.983^∗^		18.913^∗^	
∆*R*^2^	0.074		0.036	
∆*F*	—		3.088	

Notes: ^1^*B* is the unstandardized coefficient indicating the change in the dependent variable associated with a single unit of change in the independent variable. ^∗^*p* < 0.001.
